# Fetal Gender and Several Cytokines Are Associated with the Number of Fetal Cells in Maternal Blood – An Observational Study

**DOI:** 10.1371/journal.pone.0106934

**Published:** 2014-09-04

**Authors:** Jacob Mørup Schlütter, Ida Kirkegaard, Olav Bjørn Petersen, Nanna Larsen, Britta Christensen, David M. Hougaard, Steen Kølvraa, Niels Uldbjerg

**Affiliations:** 1 Department of Obstetrics and Gynecology, Aarhus University Hospital, Skejby, Aarhus, Denmark; 2 Department of Clinical Biochemistry and Immunology, Statens Serum Institut, Copenhagen, Denmark; 3 FCMB ApS, Vejle, Denmark; 4 Department of Clinical Genetics, University of Southern Denmark, Vejle Hospital, Vejle, Denmark; Gustave Roussy, France

## Abstract

**Objective:**

To identify factors influencing the number of fetal cells in maternal blood.

**Methods:**

A total of 57 pregnant women at a gestational age of weeks 11–14 were included. The number of fetal cells in maternal blood was assessed in 30 ml of blood using specific markers for both enrichment and subsequent identification.

**Results:**

Participants carrying male fetuses had a higher median number of fetal cells in maternal blood than those carrying female fetuses (5 vs. 3, *p = *0.04). Certain cytokines (RANTES, IL-2 and IL-5) were significantly associated with the number of fetal cells in maternal blood.

**Conclusion:**

The number of fetal cells in maternal blood is associated with certain cytokines and fetal gender.

## Introduction

Screening for fetal chromosome aneuploidies in high risk pregnancies has for decades been offered in many countries. Until now the sampling of fetal material for this purpose has been done by invasive procedures such as chorionic villus sampling or amniocentesis, which carry a 0.5–2% risk of unintended termination of the pregnancy [1].

Due to this risk it has long been of great interest that both circulating fetal cells and cell-free fetal DNA can be found in maternal blood [2], [3]. It has been envisioned that these fetal compounds can be utilized for a non-invasive and therefore risk-free prenatal diagnostic procedure. The development of such procedures has, however, been hampered by the fact that both cell-free fetal DNA and circulating fetal cells are very rare in blood samples from pregnant women [4], [5]. Furthermore, both cell-free fetal DNA and fetal cells are found among excessive amounts of both maternal cell-free DNA (5–10 fold excess) and maternal blood cells (10E8–10E9 fold excess). The vast excess of maternal cells may be overcome by antibody-based fetal cell enrichment since different cell types express different cell type surface epitopes. This is in contrast to cell-free fetal DNA, which is biologically and chemically very similar to cell-free maternal DNA, making it difficult to obtain efficient enrichment of the fraction of cell-free fetal DNA. In spite of these problems both cell-free fetal DNA and circulating fetal cells have been repeatedly targeted for a non-invasive prenatal diagnostic procedure. For the cell-free fetal DNA several strategies based on the initial enrichment of cell-free fetal DNA based on methylation differences have been tried but with only modest success [6], [7]. An alternative approach is to measure relative amounts of chromosome 21 DNA sequences, for example, compared to DNA sequences from reference chromosomes without prior enrichment and then look for a more modest increase in chromosome 21 sequences in trisomic cases. This approach has been tried in combination with different ways of doing the DNA quantifications, but so far only molecular counting based on next generation sequencing has had commercial success [8]–[10]. The main drawback of this approach is, however, that it is still not a diagnostic test since patients with positive results will still need an invasive procedure to finally confirm a trisomic fetus.

In regard to fetal cells the main problem has been - due to their extreme rarity - to define fetal cell specific epitopes that can be used both for initial enrichment as well as for subsequent identification of fetal cells. It has been suggested that lymphoblasts, erythroblasts and trophoblasts all circulate in maternal blood. Of these, lymphoblasts have been shown to be unfit for prenatal diagnosis [11]: regarding erythroblasts, there are findings suggesting that this fetal cell type is normally very rare in maternal blood [12], [13]. Nevertheless, several groups have tried to isolate these fetal cell types from maternal blood with a number of different antibodies, but the procedures that have been suggested to date have not survived more detailed testing in clinical settings [13]–[17] most likely because the antibodies used for enrichment were suboptimal.

Based on the uncertainty about the exact fetal cell types in maternal blood, some years ago we initiated a project to identify fetal cell type(s) [18]. In this project we collected blood samples at a gestational age of 11–14 weeks from women carrying a male fetus. We initially enriched these blood samples for fetal cells by removing a large fraction of maternal cells, followed by smearing the remaining blood cells on slides. We subsequently performed X- and Y-chromosome FISH on located male cells by automated scanning. These male cells that we considered fetal were then retrieved by laser microdissection, and mRNA was isolated. We subsequently performed expression array analysis using a stem cell array platform and analyzed the expression profiles compared to maternal blood cell profiles [19]. Among the genes significantly overexpressed in fetal cells, over half had important functions in the placenta, and about 25% could be associated with extravillous and/or endovascular trophoblasts [19]. We therefore formulated the hypothesis that a major fraction of the fetal cells in maternal blood were either extravillous and/or endovascular trophoblasts [19]. Based also on known expression patterns in these cells, we then tested a number of antibodies for their ability to enrich these fetal cells, and finally selected an antibody against CD105 as the most efficient. Subsequently, we selected cyto-keratins as a suitable marker for immunohistochemistry based identification of extravillous and endovascular trophoblasts and tested this combination of markers for fetal cell specificity on a series of fetal cells identified by the marker combination as fetal cells in maternal blood [20]. Candidate fetal cells were subsequently also analyzed by X- and Y-chromosome FISH. This series demonstrated a 96% fetal cell specificity of our marker combination on enriched cell fractions [20].

Given this high fetal cell specificity combined with well-established parameters of FISH errors, we could estimate for trisomy 21 that as few as 5–10 successfully FISHed fetal cells would be sufficient to perform a prenatal diagnosis. Our data, however, showed that among healthy pregnant women there is a substantial variation in the number of cytokeratin-7 positive fetal cells in maternal blood and that approximately 10% of pregnant women exhibit no isolated fetal cells at all when the procedure is done on 30 ml blood.

A viable method for non-invasive prenatal diagnosis based on circulating isolated fetal cells in maternal blood therefore requires a higher yield of isolated fetal cells for the analysis.

As a first step in increasing the yield, we have investigated the degree of variation in isolated fetal cells in maternal blood among pregnant women and correlated these variations to a number of clinical parameters such as placental volume, number of prior pregnancies [21], birth weight, nuchal translucency, pregnancy-associated plasma protein-A (PAPP-A), free beta human chorionic gonadotropin (free β-hCG), gestational age at blood sampling [22], concentrations of selected cytokines in maternal blood, fetal gender [23], maternal age and BMI.

## Materials and Methods

### Inclusion and cohort description

A total of 59 pregnant women at a gestational age of 11^+2^–13^+6^ weeks were included in this study. All participants signed an informed consent form. The participants were recruited at the elective first-trimester screening program offered to all pregnant women at Aarhus University Hospital, Denmark. One participant was excluded because of a twin pregnancy and another due to later miscarriage, leaving 57 pregnant women in the final study population.

In a separate series 12 patients were selected based on their isolated fetal cells counts at a gestational age of 11–14 weeks. Seven had ≥10 isolated fetal cells in 30 ml of maternal blood, and five had 0 isolated fetal cells. These patients had their placenta volume estimated by 3D ultrasound during their second trimester anomaly scan at week 19.

The study was approved by the local Danish Scientific Ethical Committee (project identification number S-20070045) and the Danish Data Protection Agency (2008-58-0035).

### Blood samples

30 ml blood samples were drawn immediately after ultrasound examination and prior to any invasive procedures. All blood samples were collected in heparinized tubes and immediately processed.

For isolated fetal cell counts 30 ml of maternal blood was fixed in PFA and maternal red blood cells lysed in Triton X-100. After pelleting of the remaining nucleated cells these were permeabilized by treatment of the cells with ice-cold methanol followed by centrifugation and washing. Cells were finally resuspended in MACS buffer (PBS, 0,5% BSA, 2 mM EDTA, Miltenyi, Germany). Fetal cells were then enriched by MACS using a combination of CD105 and CD141 microbeads (Miltenyi) and pre-washed MS columns (Miltenyi) all according to standard Miltenyi technology. The enriched cell fraction was subsequently smeared onto FLEX IHC slides (DAKO), using 2–7 slides depending on the number of maternal cells in the fraction. Slides were finally air-dried (overnight) before antibody staining by incubation for 7 minutes at 60 C. Slides were then fixed for 10 minutes in 2% PFA in PBS, rinsed and finally incubated for 30 minutes with Image-iT FX signal enhancer (Invitrogen). After rinsing and blocking in standard blocking buffer, slides were incubated for 30 minutes with a mixture of cytokeratin 7 (DAKO), pan cytokeratin (Sigma) and cytokeratin 8/18 (Invitrogen) antibodies all diluted 1∶100 in the blocking buffer. After rinsing, slides were then incubated for 30 minutes with Alexa Fluor 488 rabbit anti-mouse IgG (component A, Invitrogen) diluted 1∶200 in the blocking buffer. After another rinse, slides were incubated for 30 minutes with Alexa Fluor 488 goat anti-rabbit IgG (component B, Invitrogen) also diluted 1∶200 in the blocking buffer. Finally, slides were rinsed again and then post-fixed in 2% PFA in PBS, washed and mounted in Vectashield with DAPI (Vector Laboratories).

Positively stained fetal cells were located by fluorescence microscopy either “by hand” or by using automated scanning on a Metasystem scanner equipped with an in-house developed classifier.

Gender determination by XY-FISH on fetal cells found by this marker combination has been shown to correlate 100% with a gender determination obtained by analysis of cell-free fetal DNA in plasma when more than three fetal cells were found [20].

### Clinical parameters

Variables from the ultrasound examinations in the antenatal screening program (nuchal translucency, gestational age determined by crown-rump length (CRL)), as well as the results on serum markers (PAPP-A and free β-hCG), pre-eclampsia, preterm birth, birth weight and chromosomal abnormalities were subsequently obtained from medical databases. Maternal age and BMI, as well as information about prior pregnancies, were obtained from patients at the time of blood sampling. All scans were performed by sonographers certified by The Fetal Medical Foundation, London, UK.

### Cytokines

Concentrations of selected cytokines were determined in serum using duplicates and Luminex xMAP technology with a multiplex assay essentially as described by Skogstrand et al. 2005 [24]. The following cytokines were assessed IL-6Ra, IFN-y, TNF-R1, IL-17, IL-18, MIP-1a, MMP-9, RANTES, BDNF, GM-CSF, NT-4, NT-3, MCP-1, IL-1b, IL-2, IL-4, IL-5, IL-6, IL-8, IL-12, MIF, TREM-1, TNF-a, TNF-b, TGF-b, IL-10. As the distribution among healthy pregnant women is unknown, values were chosen to obtain maximum discrimination between lower and higher numbers of fetal cells for all cytokines. For cytokines where differences were found, the groups (high and low levels) were divided at 8 pg/ml for IL-5, 400 pg/ml for IL-2 and above 100000 pg/ml for RANTES.

### 3D placental volume estimation

3D ultrasound scans were performed on selected women at their week-19 anomaly screening using GE Voluson E8. All 3D ultrasound scans were performed by Olav Bjørn Petersen. The 3D placenta volume determination was performed offline (4D View version 9.1, GE Healthcare, Milwaukee, WI, USA). Placenta volumes were calculated using VOCAL. VOCAL II was selected, and the Define Contour: Manual option was used with 15 degrees of rotation, creating 12 slides in which the contours around the placenta were drawn manually.

First, the placenta was identified by shape and texture. The contour was drawn between the placenta and uterus where a texture change was observed. After the manual trace, the contour was fine-tuned using the 3D shell picture and the pictures in the two other plans as reference. All 3D placenta volume calculations were first done by Jacob Mørup Schlütter and then confirmed by Niels Uldbjerg.

### Statistics

Statistical analyses were performed with Stata/IC version 11.1 for Mac (64-bit Intel), StataCorp. The level of 5 percent significance was chosen. Non-parametric statistical analyses were used including Mann-Whitney-Wilcoxon test, Spearmann’s rho and Fishers Exact Test.

## Results

The 57 women participating in the study had a median age of 29 years (Range: 20–40) and a median BMI of 22.2 kg/cm^2^ (IQR: 20.1–24.9). The median gestational age at blood sampling was 12^+6^ weeks (IQR: 12^+4^–13^+0^) (Table 1). Five women had an ethnic background other than Caucasian.

**Table 1:** The median and 25–75th percentile of different continuous variables and their correlation isolated fetal cells in 30 ml maternal blood.

Continuous variables				
	n	Median	IQR	*p* (Spearmann’s rho)
BMI	57	22.2 kg/cm^2^	20.1–24.9 kg/cm^2^	0.41
PAPP-A	57	0.957 MoM	0.646–1.33 MoM	0.84
Free β-hCG	57	1.101 MoM	0.775–1.608 MoM	0.78
Nucheal translucency	57	1.7 mm	1.4–1.9 mm	0.92
Maternal age	57	29 years	27–32 years	0.70
Gestational age	57	90 days	88–91 days	0.69
Birth weight	57	3540 g	3280–3870 g	0.59

IQR: 25–75 percentiles; BMI: Body mass index; PAPP-A: Pregnancy-associated plasma protein-A; free β-hCG: free beta-human chorionic gonadotropin; MoM: Multiple of the median.

**Figure 1:**
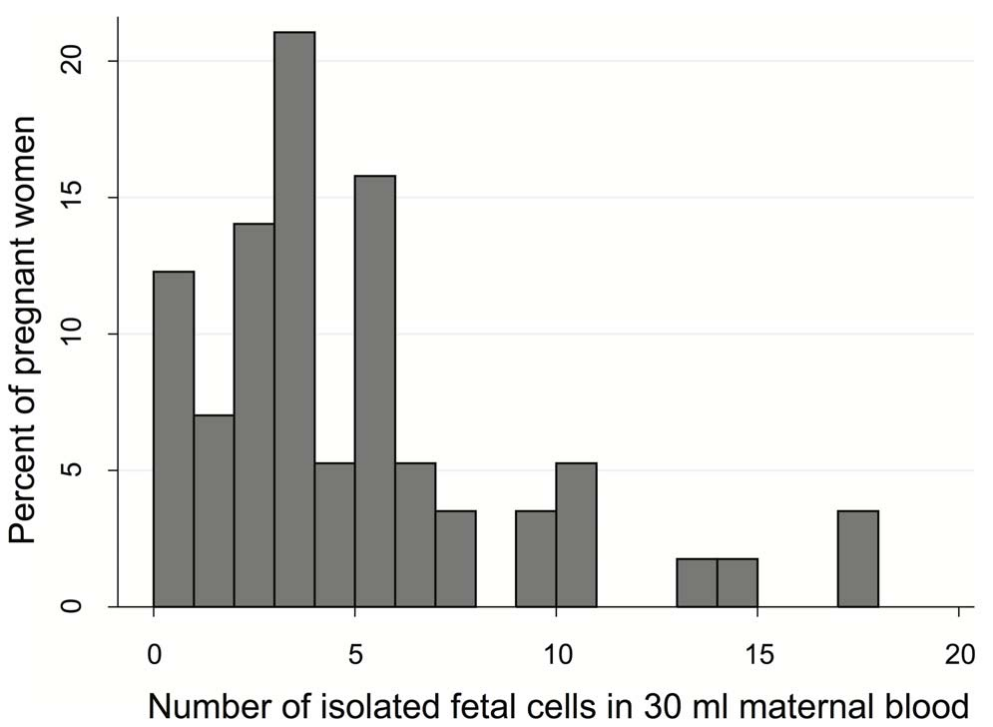
Distribution of the number of isolated fetal cells in 30 ml blood in 57 pregnant women at a gestational age of 11–14 weeks.

The median number of fetal cells per 30 ml maternal blood was 3, the 10^th^ and 90^th^ percentiles were 0 and 10, and the maximum was 18 (Figure 1). In seven of the 57 women no fetal cells were found (12%).

**Figure 2:**
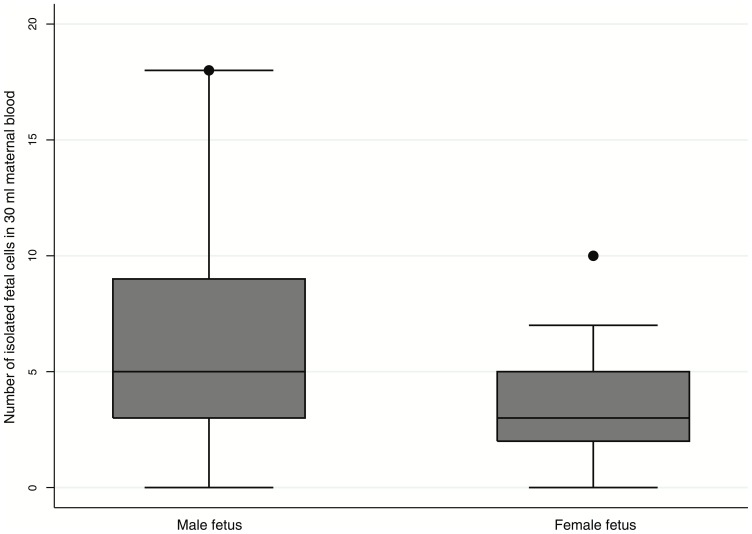
The number of isolated fetal cells in 30 ml of blood among 29 pregnant women with male fetuses and 28 women with female fetuses; *p = *0.04 (Mann-Whitney-Wilcoxon).

Pregnant women who carried a male fetus had a significantly higher number of fetal cells (median: 5 vs. 3, *p = *0.04) compared to those carrying a female fetus (Table 2, Figure 2).

**Table 2:** The number of isolated fetal cells in 30 ml maternal blood in relation to fetal gender and multipara.

Binominal variables	Yes		No		
	n	Median number ofisolated fetal cells (IQR)	n	Median number ofisolated fetal cells (IQR)	*p* (Mann-Whitney-Wilcoxon)
Fetal gender (male)	29	5 (3–9)	28	3 (2–5)	0.04
Multipara	27	3 (1–5)	30	3.5 (2–6)	0.31

IQR: IQR: 25–75 percentiles; Multipara: Parity of at least one.

**Figure 3:**
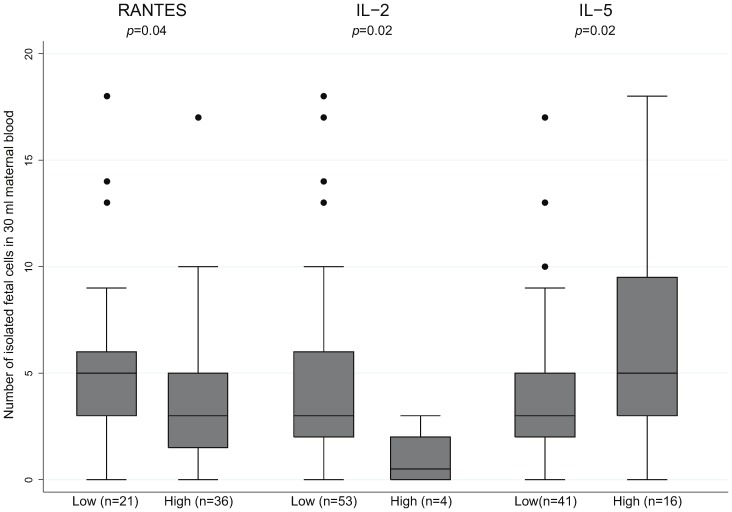
The number of isolated fetal cells in 30 ml of blood in relation to low or high levels of different selected cytokines. Three of the cytokines (RANTES, IL-2 and IL-5) had a significant association with the number of extravillous trophoblasts in maternal blood.

Increased serum concentrations of RANTES and IL-2 associated significantly with a lower number of fetal cells (Table 3 and Figure 3), and increased IL-5 concentration was significantly associated with a high number of fetal cells (Table 3 and Figure 3). The remaining 23 cytokines showed no significant association with the number of fetal cells (results not shown).

**Table 3:** The number of isolated fetal cells in 30 ml maternal blood in relation to selected cytokines.

Selected cytokines	Low levels		High levels		
	n	Median number ofisolated fetal cells (IQR)	n	Median number ofisolated fetal cells (IQR)	*p* (Mann-Whitney-Wilcoxon)
RANTES	21	5 (3–6)	36	3 (1.5–5)	0.04
IL-2	53	3 (2–6)	4	0.5 (0–2)	0.02
IL-5	41	3 (2–5)	16	5 (3–9.5)	0.02

IQR: 25–75th percentiles.

**Figure 4:**
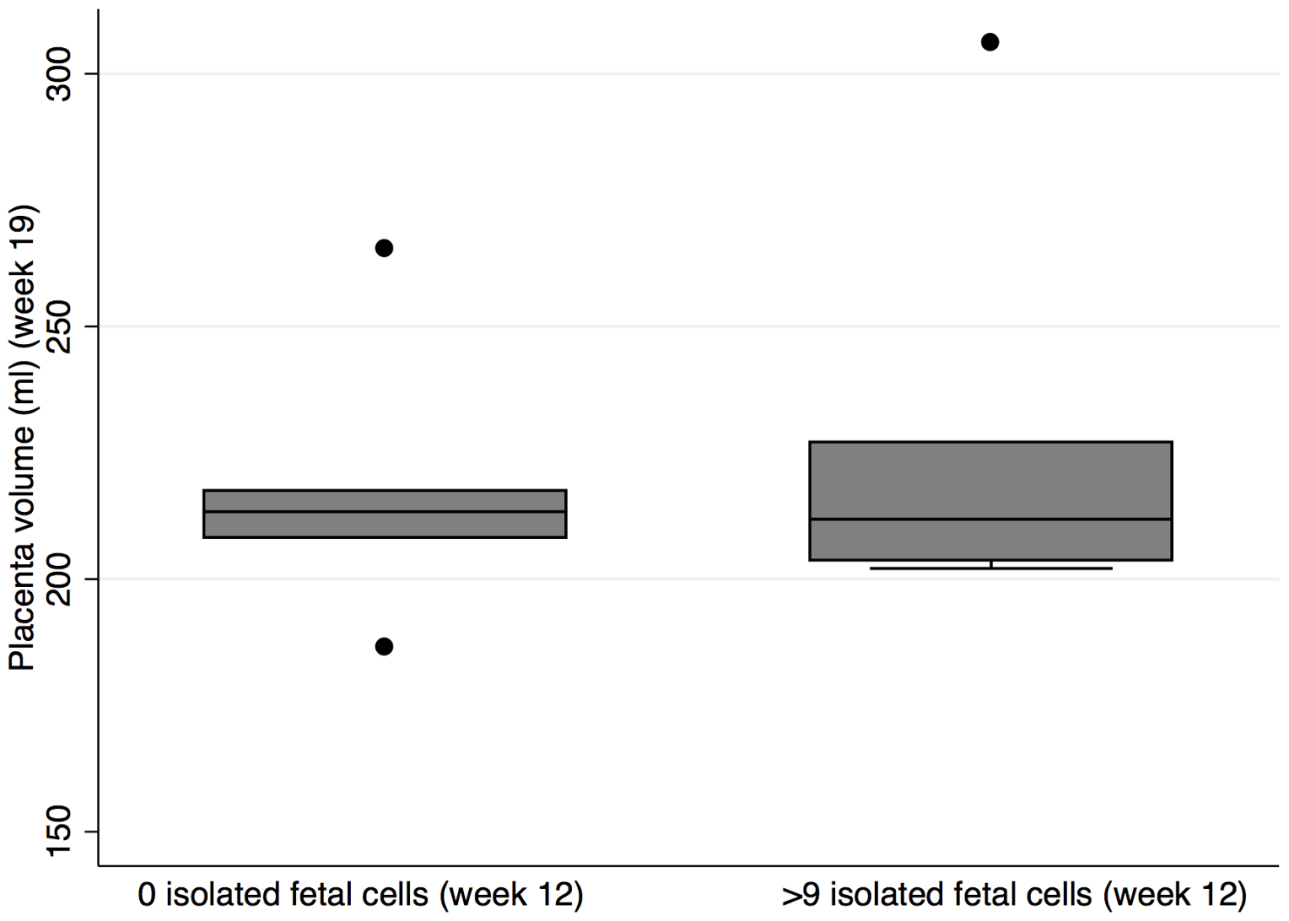
The estimated placenta size in week 19 in two groups with either a high number of isolated fetal cells (n = 7) or a low number of isolated fetal cells (n = 5) in 30 ml of maternal blood at a gestational age of 11–14 weeks, *p = *0.94 (Mann-Whitney-Wilcoxon).

We found no difference when comparing placenta size at the second-trimester anomaly ultrasound scan in seven pregnant women who had ≥10 isolated fetal cells in the blood sample compared to five pregnant women who had no isolated fetal cells in the blood sample (Figure 4).

No associations were found between the number of fetal cells and prior pregnancies, BMI, PAPP-A, free β-hCG, nucheal translucency, maternal age, gestational age or birth weight (Table 1).

The five participants who subsequently developed pre-eclampsia had a median of 5 isolated fetal cells in 30 ml maternal blood (IQR: 3–5) whereas those who did not had a median of 3 (IQR: 2–5.5; *p = *0.62). One participant delivered preterm (number of isolated fetal cells in 30 ml maternal blood: 1), and none gave birth to a child with chromosomal abnormalities.

## Discussion

Although it has been known for many years that there are fetal cells circulating in the maternal blood stream during pregnancy, a closer characterization of this phenomenon has been hampered by the scarcity of these cells combined with the fact that the fetal cell type was unknown. This lack of knowledge of fetal cell type meant that the only reliable markers available for quantification of these cells were Y chromosome specific probes that unambiguously could define cells that were derived from a male fetus.

However, it is extremely challenging to find very rare cells based on minute FISH signals; therefore, to date, few studies have performed simple quantifications on a limited series [5], [16], [22].

We have, however, recently obtained evidence that the circulating fetal cells are extravillious trophoblasts and have defined two markers that in combination exhibit a high degree of specificity for fetal cells in maternal blood. The markers are CD105 for fetal cell enrichment, combined with the immunofluorescent stain of cytokeratins.

Using these markers we have in the present communication studied the possible association between the number of isolated fetal cells in maternal blood and biological and clinical parameters. The important finding is that the median number of isolated fetal cells in 30 ml maternal blood is associated with fetal gender and serum levels of a subset of cytokines.

We find that the demonstration of a higher number of isolated fetal cells in 30 ml maternal blood of male pregnancies warrants further investigation. Fetal gender has been shown to be associated with diseases with known immunological pathologies such as pre-eclampsia, premature placental abruption and the risk of recurrent abortion [23], [25], [26], suggesting that fetal gender could be of importance to immunological responses during pregnancy. These associations could be explained by increased amounts of fetal cells circulating in maternal blood with a higher chance of developing an immunological response.

We found that a high level of RANTES or IL-2 was significantly associated with a lower number of fetal cells. Interestingly, increased levels of RANTES in amniotic fluid is associated with intrauterine infection in women delivering preterm [27] and an increased level of IL-2 is seen in the maternal blood of women with pre-eclampsia [28]. Our findings could therefore be explained by recent inflammation, which could cause the immune system to clear isolated fetal cells at an increased rate. We currently have no theory explaining why a high level of IL-5 is associated with a higher number of isolated fetal cells. However, it has been shown that a high level of IL-5 in maternal serum is associated with preterm delivery [29]. Earlier it has also been suggested that fetal mononuclear cells in maternal blood and the degree of HLA sharing within the family are associated [30]. This also emphasizes the importance of the immune system in the number of fetal cells circulating maternal blood.

Based on the specificity of 96% mentioned earlier, it can be calculated that at least five fetal cells successfully FISHed with a chromosome 21 probe are necessary to perform a reliable prenatal diagnosis for trisomy 21 on a maternal blood sample. This number was unfortunately only achieved in 40% of the participants. It is therefore extremely important to increase the number of fetal cells recovered. Considering that it has been estimated that there are 1–2 fetal cells per ml maternal blood based on X- and Y-chromosome FISH on non-enriched cell fractions [22], one way to proceed is to increase the yield of fetal cells in the enrichment, which we are presently working on. A first step in this process is to elucidate which factors affect the number of fetal cells in maternal blood. In the present communication we have demonstrated that the number of fetal cells is associated with fetal gender and a number of cytokines. These findings might inspire future studies into factors that might influence the number of fetal cells in maternal blood.

## Supporting Information

Table S1 **Raw data on which analysis were performed.**
(DOCX)
