# Supplementary material for: Fetal Gender and Several Cytokines Are Associated with the Number of Fetal Cells in Maternal Blood – An Observational Study
Source: PLoS One. 2014 Sep 4;9(9):e106934. doi: 10.1371/journal.pone.0106934 (PMC4154776; doi:10.1371/journal.pone.0106934)
Supplement: Table S1 — Raw data on which analysis were performed. (DOCX) [file pone.0106934.s001.docx]

Table S1: Raw data

| Isolated fetal cells | Maternal Age (years) | Gestational age (days) | Nuchal translucency (mm) | Free β-hCG (MoM) | PAPP-A (MoM) | BMI (kg/cm^2^) | Parity | Birth weight (g) | Gender | IFN-y (pg/mL) | MIP-1a (pg/mL) | MMP-9 (pg/mL) | RANTES (pg/mL) | BDNF (pg/mL) | GM-CSF (pg/mL) | NT-4 (pg/mL) | NT-3 (pg/mL) | MCP-1 (pg/mL) | IL-1b (pg/mL) | IL-2 (pg/mL) | IL-4 (pg/mL) | IL-5 (pg/mL) | IL-6 (pg/mL) | IL-6ra (pg/mL) | IL-8 (pg/mL) | IL-10 (pg/mL) | IL-12 (pg/mL) | IL-17 (pg/mL) | IL-18 (pg/mL) | MIF (pg/mL) | TREM-1 (pg/mL) | TNF-a (pg/mL) | TNF-b (pg/mL) | TNF-R1 (pg/mL) | TGF-b (pg/mL) | Pre-eclampsia | Preterm Birth | Placenta size (ml) |
| --- | --- | --- | --- | --- | --- | --- | --- | --- | --- | --- | --- | --- | --- | --- | --- | --- | --- | --- | --- | --- | --- | --- | --- | --- | --- | --- | --- | --- | --- | --- | --- | --- | --- | --- | --- | --- | --- | --- |
| 0 | 29 | 85 | 1.3 | 1.03 | 1.10 | 24.8 | Nullipara | 3750 | XY | 4 | 273 | 5000000 | 160000 | 45883 | 10 | 4 | 45 | 236 | 70 | 1310 | 4 | 4 | 4 | 95202 | 4 | 211 | 9 | 9 | 327 | 1338 | 488 | 4 | 145 | 1831 | 39 | No | No | N/A |
| 0 | 37 | 92 | 1.8 | 1.42 | 0.80 | 32.7 | Multipara | 4420 | XX | 4 | 239 | 5000000 | 160000 | 28293 | 10 | 4 | 4 | 218 | 29 | 4 | 4 | 4 | 4 | 51219 | 4 | 4 | 4 | 4 | 743 | 98 | 488 | 4 | 10 | 1515 | 39 | No | No | 265.318 |
| 0 | 37 | 91 | 1.4 | 2.26 | 0.86 | 22.9 | Multipara | 4210 | XX | 4 | 595 | 318056 | 160000 | 8130 | 10 | 4 | 70 | 10 | 42 | 4 | 4 | 4 | 4 | 63436 | 4 | 144 | 4 | 4 | 349 | 621 | 488 | 4 | 10 | 1385 | 39 | No | No | N/A |
| 0 | 29 | 91 | 1.9 | 1.17 | 0.51 | 22.3 | Multipara | 4550 | XY | 15 | 2195 | 5000000 | 160000 | 11016 | 10 | 4 | 79 | 266 | 62 | 492 | 4 | 20 | 14 | 83661 | 4 | 877 | 80 | 4 | 532 | 1211 | 17200 | 4 | 490 | 2194 | 39 | No | No | 217.495 |
| 0 | 24 | 91 | 2.2 | 1.27 | 0.63 | 19.7 | Nullipara | 3130 | XX | 4 | 1853 | 334015 | 13089 | 15797 | 10 | 4 | 4 | 10 | 29 | 4 | 4 | 4 | 18 | 79919 | 4 | 147 | 19 | 4 | 413 | 840 | 3955 | 4 | 200 | 1407 | 39 | No | No | 186.352 |
| 0 | 28 | 92 | 2.8 | 1.08 | 1.11 | 21.5 | Multipara | 3440 | XY | 27 | 182 | 5000000 | 160000 | 19048 | 10 | 4 | 4 | 369 | 52 | 4 | 4 | 4 | 4 | 77594 | 4 | 635 | 58 | 4 | 273 | 1342 | 488 | 4 | 10 | 1112 | 39 | No | No | 213.336 |
| 0 | 32 | 93 | 1.6 | 2.45 | 1.40 | 20.1 | Multipara | 3980 | XX | 4 | 106 | 5000000 | 160000 | 22259 | 10 | 4 | 4 | 833 | 11 | 4 | 4 | 4 | 4 | 41980 | 4 | 609 | 4 | 4 | 365 | 98 | 488 | 4 | 10 | 1515 | 39 | No | No | 207.951 |
| 1 | 26 | 87 | 1.5 | 1.80 | 0.61 | 20.5 | Nullipara | 3725 | XY | 41 | 303 | 5000000 | 160000 | 15620 | 10 | 4 | 4 | 546 | 114 | 4 | 4 | 4 | 4 | 76852 | 4 | 137 | 28 | 4 | 496 | 477 | 488 | 614 | 132 | 1859 | 39 | Yes | No | N/A |
| 1 | 30 | 95 | 2.0 | 0.53 | 1.06 | 18.6 | Multipara | 3760 | XY | 27 | 189 | 5000000 | 160000 | 18225 | 10 | 4 | 67 | 556 | 63 | 1119 | 4 | 4 | 432 | 83779 | 38 | 162 | 505 | 18 | 574 | 2020 | 28269 | 4 | 181 | 2137 | 334 | No | No | N/A |
| 1 | 29 | 85 | 1.1 | 0.64 | 0.82 | 25.1 | Multipara | 3350 | XX | 4 | 180 | 5000000 | 160000 | 52757 | 44 | 8 | 4 | 294 | 49 | 4 | 4 | 4 | 13 | 79414 | 4 | 157 | 4 | 9 | 433 | 3541 | 488 | 4 | 60 | 1565 | 39 | No | No | N/A |
| 1 | 30 | 86 | 1.2 | 0.59 | 0.49 | 17.9 | Multipara | 2590 | XY | 4 | 187 | 300357 | 5731 | 13600 | 10 | 13 | 4 | 464 | 76 | 4 | 4 | 4 | 9 | 77077 | 4 | 562 | 10 | 4 | 710 | 98 | 488 | 4 | 157 | 1464 | 39 | No | Yes | N/A |
| 2 | 33 | 83 | 1.3 | 1.11 | 1.53 | 23.4 | Multipara | 3530 | XX | 4 | 157 | 5000000 | 160000 | 27927 | 10 | 4 | 4 | 319 | 69 | 4 | 4 | 8 | 4 | 60905 | 4 | 152 | 177 | 4 | 358 | 1351 | 488 | 4 | 42 | 1823 | 39 | No | No | N/A |
| 2 | 26 | 90 | 1.5 | 1.99 | 1.05 | 26.1 | Nullipara | 3380 | XX | 4 | 385 | 5000000 | 160000 | 21577 | 10 | 10 | 95 | 10 | 45 | 4 | 4 | 4 | 12 | 77491 | 4 | 321 | 4 | 4 | 521 | 3425 | 488 | 4 | 109 | 1773 | 39 | No | No | N/A |
| 2 | 31 | 88 | 1.7 | 0.37 | 1.38 | 25.0 | Nullipara | 3600 | XY | 4 | 372 | 5000000 | 160000 | 32545 | 10 | 4 | 249 | 754 | 62 | 4 | 4 | 8 | 4 | 73977 | 4 | 189 | 4 | 4 | 355 | 1637 | 488 | 4 | 72 | 1850 | 39 | No | No | N/A |
| 2 | 32 | 90 | 2.6 | 0.72 | 0.94 | 20.0 | Nullipara | 3880 | XX | 4 | 212 | 217786 | 160000 | 9431 | 10 | 4 | 4 | 10 | 30 | 4 | 4 | 4 | 4 | 35903 | 4 | 4 | 4 | 4 | 279 | 98 | 488 | 4 | 10 | 1231 | 39 | No | No | N/A |
| 2 | 27 | 91 | 1.4 | 1.88 | 1.12 | 21.8 | Nullipara | 3040 | XX | 4 | 299 | 5000000 | 160000 | 19607 | 10 | 18 | 117 | 772 | 58 | 4 | 4 | 16 | 4 | 91933 | 4 | 184 | 31 | 34 | 355 | 213 | 488 | 4 | 264 | 1773 | 123 | No | No | N/A |
| 2 | 30 | 91 | 1.4 | 2.47 | 0.96 | 21.1 | Multipara | 3090 | XX | 4 | 641 | 199153 | 6931 | 16008 | 10 | 15 | 4 | 525 | 54 | 4 | 4 | 4 | 22 | 80760 | 4 | 200 | 27 | 4 | 417 | 752 | 488 | 4 | 98 | 1403 | 92 | No | No | N/A |
| 2 | 33 | 90 | 1.7 | 1.10 | 1.18 | 24.9 | Nullipara | 3940 | XX | 4 | 169 | 5000000 | 160000 | 20368 | 10 | 4 | 4 | 330 | 11 | 4 | 4 | 4 | 4 | 92021 | 4 | 182 | 4 | 10 | 412 | 98 | 488 | 4 | 41 | 1373 | 39 | No | No | N/A |
| 2 | 30 | 85 | 1.4 | 0.92 | 0.42 | 27.9 | Nullipara | 3535 | XX | 4 | 160 | 5000000 | 160000 | 12171 | 10 | 8 | 4 | 635 | 37 | 4 | 4 | 4 | 4 | 78169 | 4 | 89 | 4 | 4 | 429 | 98 | 488 | 4 | 108 | 1205 | 39 | No | No | N/A |
| 3 | 27 | 90 | 2.0 | 1.36 | 1.12 | 31.9 | Nullipara | 2990 | XY | 4 | 175 | 173538 | 6949 | 10917 | 10 | 4 | 4 | 173 | 4 | 4 | 4 | 13 | 4 | 65959 | 4 | 115 | 4 | 4 | 373 | 98 | 488 | 4 | 114 | 1203 | 39 | Yes | No | N/A |
| 3 | 32 | 91 | 2.1 | 1.09 | 1.12 | 24.3 | Multipara | 3950 | XX | 4 | 214 | 314341 | 6513 | 16126 | 10 | 16 | 4 | 634 | 73 | 4 | 4 | 4 | 45 | 47832 | 4 | 276 | 41 | 53 | 473 | 3197 | 488 | 4 | 244 | 1468 | 39 | No | No | N/A |
| 3 | 36 | 90 | 1.9 | 1.75 | 1.98 | 22.2 | Multipara | 3100 | XX | 4 | 251 | 302746 | 6115 | 16016 | 10 | 4 | 215 | 10 | 51 | 4 | 4 | 4 | 4 | 78432 | 4 | 205 | 14 | 4 | 209 | 2236 | 488 | 4 | 10 | 1111 | 39 | No | No | N/A |
| 3 | 29 | 85 | 1.8 | 1.28 | 1.64 | 27.2 | Nullipara | 3630 | XY | 4 | 306 | 5000000 | 160000 | 62443 | 10 | 11 | 209 | 212 | 111 | 4 | 4 | 9 | 4 | 168831 | 4 | 245 | 23 | 19 | 481 | 2708 | 488 | 4 | 191 | 2533 | 39 | No | No | N/A |
| 3 | 27 | 92 | 2.0 | 0.49 | 0.82 | 32.7 | Multipara | 3070 | XX | 4 | 1199 | 5000000 | 160000 | 19926 | 10 | 11 | 4 | 614 | 44 | 4 | 4 | 4 | 35 | 88027 | 4 | 991 | 66 | 17 | 493 | 1261 | 488 | 4 | 153 | 1784 | 39 | No | No | N/A |
| 3 | 27 | 97 | 2.1 | 0.68 | 0.66 | 20.8 | Nullipara | 3560 | XX | 4 | 176 | 332131 | 7098 | 15796 | 10 | 4 | 192 | 10 | 47 | 4 | 4 | 4 | 33 | 78933 | 4 | 196 | 20 | 27 | 280 | 98 | 488 | 4 | 265 | 1624 | 39 | No | No | N/A |
| 3 | 27 | 88 | 1.8 | 0.60 | 0.53 | 19.5 | Nullipara | 3590 | XX | 4 | 165 | 368897 | 6924 | 16699 | 10 | 9 | 4 | 926 | 205 | 4 | 4 | 8 | 20 | 78810 | 4 | 2166 | 17 | 4 | 239 | 2911 | 488 | 4 | 10 | 1813 | 39 | No | No | N/A |
| 3 | 26 | 92 | 1.7 | 1.31 | 2.35 | 19.4 | Multipara | 2930 | XX | 4 | 242 | 176484 | 160000 | 6061 | 10 | 4 | 4 | 259 | 36 | 1089 | 4 | 4 | 11 | 44131 | 9 | 1669 | 46 | 4 | 585 | 1954 | 4975 | 160 | 20 | 1524 | 39 | No | No | N/A |
| 3 | 35 | 94 | 1.9 | 1.01 | 1.13 | 22.2 | Multipara | 3540 | XY | 4 | 148 | 5000000 | 160000 | 20877 | 10 | 4 | 4 | 268 | 16 | 4 | 4 | 4 | 4 | 53995 | 4 | 104 | 10 | 4 | 381 | 98 | 488 | 4 | 10 | 698 | 39 | No | No | N/A |
| 3 | 29 | 88 | 1.4 | 1.03 | 2.17 | 21.6 | Nullipara | 3340 | XY | 4 | 237 | 5000000 | 160000 | 48942 | 10 | 15 | 4 | 28 | 60 | 4 | 92 | 31 | 4 | 47977 | 4 | 311 | 20 | 20 | 374 | 402 | 488 | 4 | 99 | 1521 | 39 | No | No | N/A |
| 3 | 29 | 83 | 1.4 | 1.36 | 1.79 | 20.8 | Nullipara | 4230 | XX | 4 | 302 | 5000000 | 160000 | 28672 | 10 | 4 | 4 | 540 | 45 | 4 | 4 | 4 | 4 | 76649 | 4 | 107 | 18 | 4 | 198 | 659 | 488 | 4 | 36 | 1993 | 39 | No | No | N/A |
| 3 | 30 | 91 | 1.8 | 0.89 | 0.65 | 21.1 | Multipara | 3950 | XY | 14 | 325 | 5000000 | 160000 | 20573 | 10 | 11 | 144 | 392 | 70 | 4 | 4 | 10 | 44 | 45388 | 4 | 459 | 41 | 27 | 217 | 1652 | 2214 | 4 | 312 | 1547 | 39 | No | No | N/A |
| 4 | 29 | 84 | 2.0 | 1.25 | 2.03 | 24.7 | Nullipara | 2900 | XX | 4 | 185 | 5000000 | 160000 | 24106 | 10 | 4 | 8 | 452 | 74 | 4 | 4 | 4 | 4 | 107388 | 11 | 306 | 23 | 14 | 419 | 5899 | 488 | 116 | 78 | 1689 | 586 | No | No | N/A |
| 4 | 23 | 86 | 1.5 | 1.61 | 0.29 | 18.5 | Multipara | 3330 | XY | 46 | 202 | 385130 | 9553 | 4541 | 10 | 4 | 4 | 314 | 22 | 4 | 4 | 4 | 4 | 82919 | 4 | 222 | 45 | 4 | 588 | 98 | 488 | 4 | 105 | 1668 | 39 | No | No | N/A |
| 4 | 25 | 97 | 2.0 | 1.20 | 0.68 | 26.1 | Nullipara | 3420 | XY | 4 | 214 | 318163 | 5657 | 16483 | 10 | 8 | 4 | 529 | 28 | 4 | 4 | 4 | 15 | 76065 | 4 | 282 | 36 | 4 | 282 | 8663 | 488 | 4 | 160 | 1388 | 39 | No | No | N/A |
| 5 | 27 | 88 | 1.4 | 0.46 | 0.39 | 19.7 | Multipara | 3125 | XY | 11 | 176 | 193421 | 7090 | 16270 | 10 | 8 | 4 | 604 | 93 | 4 | 4 | 4 | 17 | 75470 | 4 | 253 | 38 | 42 | 285 | 98 | 488 | 4 | 137 | 1782 | 39 | Yes | No | N/A |
| 5 | 31 | 91 | 2.0 | 0.40 | 0.55 | 25.0 | Multipara | 2790 | XY | 4 | 297 | 5000000 | 160000 | 30987 | 10 | 12 | 4 | 520 | 104 | 4 | 4 | 9 | 4 | 117426 | 4 | 390 | 20 | 4 | 210 | 2693 | 488 | 4 | 20 | 1591 | 39 | No | No | N/A |
| 5 | 28 | 93 | 2.4 | 1.17 | 1.13 | 23.7 | Multipara | 3600 | XX | 4 | 149 | 5000000 | 160000 | 23910 | 10 | 4 | 4 | 247 | 39 | 4 | 4 | 4 | 4 | 79523 | 4 | 734 | 19 | 4 | 274 | 98 | 488 | 4 | 10 | 702 | 39 | No | No | N/A |
| 5 | 26 | 88 | 1.4 | 0.32 | 1.03 | 24.2 | Nullipara | 3530 | XX | 4 | 133 | 404797 | 12585 | 19450 | 10 | 4 | 4 | 311 | 285 | 4 | 4 | 12 | 14 | 69564 | 4 | 78 | 21 | 19 | 412 | 1060 | 488 | 4 | 284 | 1170 | 39 | Yes | No | N/A |
| 5 | 34 | 87 | 1.5 | 0.48 | 0.45 | 25.3 | Nullipara | 3400 | XY | 4 | 133 | 5000000 | 160000 | 19078 | 10 | 4 | 4 | 661 | 9 | 4 | 4 | 4 | 4 | 95992 | 4 | 467 | 4 | 4 | 199 | 3619 | 488 | 4 | 10 | 1577 | 39 | No | No | N/A |
| 5 | 30 | 92 | 1.7 | 1.10 | 0.46 | 19.2 | Nullipara | 3300 | XX | 4 | 227 | 5000000 | 11338 | 19391 | 10 | 4 | 4 | 85 | 4 | 4 | 4 | 12 | 4 | 48445 | 4 | 4 | 4 | 4 | 130 | 98 | 488 | 4 | 10 | 734 | 39 | No | No | N/A |
| 5 | 23 | 86 | 1.2 | 2.95 | 1.66 | 22.5 | Nullipara | 2720 | XX | 4 | 10 | 5000000 | 8522 | 18965 | 10 | 4 | 4 | 10 | 4 | 4 | 4 | 4 | 4 | 68286 | 4 | 4 | 4 | 4 | 236 | 98 | 488 | 4 | 10 | 1030 | 39 | No | No | N/A |
| 5 | 28 | 88 | 1.6 | 1.24 | 2.07 | 26.8 | Nullipara | 4100 | XY | 4 | 155 | 286478 | 160000 | 18440 | 10 | 4 | 36 | 499 | 110 | 4 | 4 | 4 | 4 | 59461 | 4 | 137 | 4 | 4 | 326 | 1543 | 488 | 4 | 142 | 1736 | 39 | No | No | N/A |
| 5 | 32 | 88 | 1.0 | 0.83 | 0.73 | 21.0 | Multipara | 3940 | XX | 4 | 151 | 5000000 | 160000 | 26152 | 10 | 4 | 4 | 223 | 66 | 4 | 4 | 4 | 4 | 76312 | 4 | 210 | 33 | 25 | 281 | 98 | 488 | 4 | 311 | 1357 | 39 | No | No | N/A |
| 6 | 29 | 91 | 1.8 | 0.67 | 0.95 | 20.2 | Nullipara | 3560 | XX | 4 | 163 | 236631 | 6650 | 16871 | 21 | 10 | 4 | 1012 | 94 | 4 | 4 | 4 | 16 | 70200 | 4 | 178 | 67 | 4 | 300 | 4421 | 488 | 4 | 28 | 1689 | 331 | No | No | N/A |
| 6 | 33 | 93 | 1.7 | 3.89 | 1.69 | 23.2 | Multipara | 3810 | XY | 4 | 198 | 5000000 | 160000 | 16743 | 10 | 4 | 4 | 334 | 72 | 108 | 4 | 17 | 4 | 59961 | 4 | 241 | 69 | 18 | 465 | 98 | 1061 | 4 | 205 | 1897 | 39 | No | No | N/A |
| 6 | 29 | 90 | 1.8 | 2.01 | 1.46 | 21.8 | Multipara | 4210 | XY | 4 | 232 | 338784 | 6979 | 16010 | 10 | 4 | 4 | 797 | 49 | 4 | 4 | 8 | 4 | 79301 | 4 | 160 | 16 | 4 | 381 | 98 | 488 | 4 | 37 | 1317 | 39 | No | No | N/A |
| 7 | 20 | 90 | 1.7 | 0.94 | 0.66 | 20.7 | Nullipara | 2780 | XX | 4 | 202 | 306235 | 6179 | 16694 | 10 | 8 | 124 | 767 | 96 | 4 | 4 | 16 | 50 | 77564 | 4 | 120 | 36 | 51 | 597 | 6384 | 488 | 4 | 191 | 1643 | 39 | No | No | N/A |
| 7 | 37 | 89 | 1.8 | 2.51 | 0.87 | 19.6 | Multipara | 4000 | XY | 4 | 238 | 5000000 | 160000 | 33371 | 10 | 4 | 4 | 393 | 61 | 4 | 4 | 4 | 4 | 106541 | 4 | 269 | 4 | 8 | 429 | 98 | 488 | 4 | 81 | 1770 | 161 | No | No | N/A |
| 9 | 32 | 89 | 1.4 | 0.45 | 1.81 | 29.9 | Nullipara | 3250 | XY | 4 | 119 | 305164 | 7159 | 17112 | 10 | 4 | 4 | 690 | 28 | 4 | 4 | 4 | 4 | 79655 | 4 | 200 | 18 | 4 | 437 | 98 | 488 | 4 | 10 | 1476 | 39 | Yes | No | N/A |
| 9 | 27 | 83 | 1.1 | 0.89 | 0.82 | 27.6 | Multipara | 4170 | XY | 4 | 615 | 5000000 | 160000 | 18658 | 31 | 4 | 55 | 1121 | 116 | 4 | 4 | 73 | 8 | 81020 | 4 | 470 | 27 | 4 | 501 | 1968 | 488 | 4 | 257 | 2763 | 39 | No | No | N/A |
| 10 | 25 | 91 | 1.7 | 0.99 | 0.64 | 19.5 | Nullipara | 3870 | XY | 4 | 541 | 5000000 | 160000 | 28289 | 10 | 4 | 172 | 10 | 70 | 4 | 4 | 9 | 4 | 65333 | 4 | 410 | 27 | 4 | 340 | 98 | 488 | 4 | 54 | 1926 | 39 | No | No | 226.968 |
| 10 | 40 | 92 | 2.4 | 0.88 | 0.92 | 24.6 | Nullipara | 3820 | XX | 25 | 283 | 5000000 | 160000 | 21428 | 10 | 4 | 60 | 852 | 83 | 4 | 4 | 9 | 29 | 61435 | 4 | 406 | 78 | 12 | 276 | 1500 | 488 | 4 | 93 | 1666 | 39 | No | No | 210.073 |
| 10 | 34 | 88 | 1.8 | 1.77 | 1.20 | 20.8 | Nullipara | 3420 | XY | 4 | 148 | 259256 | 160000 | 18847 | 10 | 4 | 4 | 261 | 54 | 4 | 4 | 4 | 83 | 78841 | 4 | 1153 | 149 | 4 | 305 | 2189 | 488 | 4 | 114 | 1562 | 39 | No | No | 211.846 |
| 13 | 32 | 96 | 2.0 | 1.53 | 1.25 | 19.0 | Nullipara | 3720 | XY | 8 | 313 | 323831 | 6474 | 16663 | 29 | 19 | 4 | 574 | 103 | 4 | 4 | 8 | 55 | 46876 | 4 | 636 | 42 | 62 | 383 | 803 | 488 | 4 | 312 | 1506 | 39 | No | No | 202.103 |
| 14 | 25 | 89 | 1.8 | 1.99 | 0.63 | 18.9 | Multipara | 3820 | XY | 4 | 10 | 149189 | 9316 | 16666 | 10 | 4 | 4 | 257 | 4 | 4 | 4 | 32 | 4 | 31916 | 4 | 4 | 4 | 4 | 315 | 98 | 488 | 4 | 10 | 558 | 39 | No | No | 203.598 |
| 17 | 29 | 85 | 1.3 | 0.77 | 0.44 | 19.5 | Multipara | 3410 | XY | 4 | 292 | 5000000 | 160000 | 28626 | 10 | 4 | 4 | 297 | 20 | 4 | 4 | 4 | 4 | 75339 | 4 | 103 | 4 | 32 | 377 | 2883 | 488 | 4 | 296 | 988 | 39 | No | No | 214.800 |
| 18 | 30 | 90 | 1.4 | 1.70 | 1.33 | 22.7 | Nullipara | 3280 | XY | 4 | 330 | 402887 | 12719 | 11473 | 10 | 4 | 294 | 375 | 45 | 4 | 4 | 13 | 4 | 46382 | 4 | 158 | 25 | 4 | 313 | 2643 | 488 | 4 | 10 | 1297 | 39 | No | No | 306.037 |
